# Management of hypoxemia in SARS-CoV-2 infection: Lessons learned from one year of experience, with a special focus on silent hypoxemia

**DOI:** 10.1016/j.jointm.2021.02.001

**Published:** 2021-03-09

**Authors:** Ricardo Serrano, Xavier Corbella, Jordi Rello

**Affiliations:** 1 Critical Care Department. Hospital de Hellín. Gerencia Atención Integrada de Hellín, Albacete 02400, Spain; 2 Interna Medicine Department, Hospital Universitari de Bellvitge-IDIBELL, L'Hospitalet de Llobregat, Barcelona 08907, Spain; 3 School of Medicine, Universitat Internacional de Catalunya, Barcelona 08017, Spain; 4 Vall d'Hebron Institute of Research, Barcelona 08035, Spain; 5 Research in Critical Care, CHU Caremeau, Nîmes 30900, France; 6 Centro de Investigación Biomédica en Red de Enfermedades Respiratorias (CIBERES), Instituto de Salud Carlos III, Madrid 28029, Spain

**Keywords:** COVID-19, Acute respiratory distress syndrome (ARDS), Pandemic, Critical care

## Abstract

Silent hypoxemia is common in patients with severe acute respiratory syndrome coronavirus 2 (SARS-CoV-2) infection. In this article, the possible pathophysiological mechanisms underlying respiratory symptoms have been reviewed, and the presence of hypoxemia without hypoxia is also discussed. The experience we have gained since the start of the Coronavirus disease 19 (COVID-19) pandemic has changed our point of view about which patients with respiratory involvement should be admitted to the intensive care unit/high-dependency unit for mechanical ventilation and monitoring. In patients with clinically well-tolerated mild to moderate hypoxemia (silent hypoxemia), regardless of the extent of pulmonary opacities found in radiological studies, the administration of supplemental oxygen therapy may increase the risk of endothelial damage. The risk of sudden respiratory arrest during emergency intubation, which could expose healthcare workers to infection, should be considered along with the risks of premature intubation. Criteria for intubation need to be revisited based on updated evidence showing that many patients with severe hypoxemia do not show increased work of breathing. This has implications in patient management and may explain in part reports of broad differences in outcomes among intubated patients.

## Highlights

• The most important issues in the care are monitoring respiratory status by direct observation and SpO2 continuous monitoring to decide whether endotracheal intubation is indicated.
• In patients with silent hypoxemia, the administration of supplemental oxygen therapy is required but no significant injury should be expected with SpO2 above 80%.
• Patients with symptomatic moderate-severe hypoxemia could benefit from the administration of supplemental oxygen through reservoir masks, or HFNC. A trial of awake prone position should be considered.
• The decision of intubation and mechanical ventilation should not be considered based on a single isolated parameter, such as hypoxemia degree or the extent of pulmonary infiltrates in imaging tests.

## Introduction

Coronavirus disease 19 (COVID-19) pandemic has proven to be a challenge in different areas of care for patients admitted to intensive care units. Application of oxygen therapies and respiratory support measures is challenging because COVID-19 differs from other viral respiratory infections (such as influenza and seasonal endemic coronavirus). Given the lack of knowledge supported by scientific evidence, medical treatment has been administered based on expert opinions. Severe acute respiratory syndrome coronavirus 2 (SARS-CoV-2) infection is associated with a peculiar form of hypoxemic respiratory failure that differs greatly from other types of lung involvement induced by bacterial respiratory infections, showing the presence of endotheliitis, thrombotic vascular events, and a delayed hyperinflammatory systemic reaction.

Hypoxia is defined as a reduction in oxygen levels in tissues. It is not measured directly by a laboratory test but is instead characterized by lactate production due to the activation of anaerobic metabolism. Hypoxemia is defined as a drop in the arterial pressure of oxygen below 80 mmHg. There are five pulmonary mechanisms that result in hypoxemia including alveolar hypoventilation, right to left shunt, diffusion–perfusion abnormality, diffusion impairment and ventilation–perfusion mismatch, These usually manifest as shortness of breath. “Silent (also referred to as “happy”) hypoxemia” has been defined as a form of hypoxia that is well tolerated by early-stage patients with no sensation of dyspnea or increased respiratory work [1]. Different methods of oxygen administration, such as the use of a high-flow nasal cannula (HFNC) [2], awake prone positioning [3,4], and noninvasive ventilation [5], have been used to improve arterial oxygenation.

SARS-CoV-2 infection is a systemic complex disease with multiple organ involvement, and respiratory alterations may be caused by several factors, including the coexistence of hyperperfused and hypoperfused areas (the relative percentage of these areas differs according to the severity and stage of the disease). Hypoxemia is mainly caused by the presence of poorly aerated areas and a low ventilation/perfusion ratio or normally aerated/hyperperfused areas. Dual-energy computed tomography (CT) scans have demonstrated the presence of lung perfusion deficits that increase dead space. The dual-energy CT pattern characteristic of SARS-CoV-2 infection with perfusion abnormalities described recently indicates that we are observing a new clinical process [6]. The respiratory distress associated with SARS-CoV-2 infection has been defined as acute respiratory distress syndrome (ARDS) associated with COVID-19 (C-ARDS) [7]. This definition may be questioned if we note that this should be considered a multisystemic process that includes both alveolar and vascular involvement. With respect to respiratory pathology, its behavior and clinical management differ depending on disease evolution. According to the Berlin definition of ARDS [8], three grades with corresponding mortality risks can be defined according to the degree of hypoxemia and opacities observed in imaging tests, once a cardiac cause has been ruled out; however, some auxiliary parameters were removed from the definition as they were not related to the probability of mortality, such as pulmonary compliance values (cutoff point, ≤40 ml/cm H_2_O) [8]. In cases of early respiratory failure caused by SARS-CoV-2, lung compliance values close to normal [9] are observed, unlike in classic cases of ARDS. However, compliance values may vary depending on the extent of the affected aerated areas, prior exposure to noninvasive ventilation or the implementation of positive end-expiratory pressure implemented, and whether a terminal phenotype is observed [10].

The temporal evaluation of silent hypoxemia in patients with COVID-19 requires a supply of inhaled oxygen at low concentration and noninvasive respiratory support using supplemental high-flow oxygen therapy through a HFNC or continuous positive airway pressure (CPAP) systems, together with awake prone positioning, if possible. Other measures are more controversial; these include the use of noninvasive ventilation systems, which may spread the virus through aerosols, and artisanal systems consisting of facial masks with positive-pressure input, which are associated with the risk of severe respiratory acidosis in chronic respiratory patients in case these systems are abruptly withdrawn accidentally or voluntarily [11].

In other types of hypoxemic respiratory failure, such as those caused by bacteria [12], [13], [14], [15], hypoxemia is the key to isolating airways through orotracheal intubation and starting invasive mechanical ventilation as there is a correlation between the degree of hypoxia and respiratory mechanics [10]. This principle is difficult to understand in cases of silent hypoxemia because clinical tolerance to hypoxia casts doubt on the benefits of using a ventilator to replace respiratory function (as well as the ventilatory parameters to be applied). The benefits of rescue techniques for refractory hypoxemia, such as prone positioning, use of extracorporeal membrane oxygenation, and administration of inhaled nitric oxide [16], remain unclear.

## Possible pathophysiological mechanism

The respiratory infection caused by SARS-CoV-2 exhibits different physio-pathological, clinical, and responses to respiratory support. A shunt effect could be suspected given the poor response to the increase in the fraction of inhaled oxygen with oxygenation. This would correspond to a greater extent with an effect derived from extensive lung involvement and an alteration in the relationship ventilation–perfusion ratio. Lung involvement is bilateral and patchy, with hyperventilated areas alternating with hypoventilated areas.

With respect to dead space, the clinical picture with COVID-19 could differ from that seen in ARDS secondary to pneumococcal pneumonia, where diffuse alveolar involvement is an impediment to the elimination of CO_2_, resulting in hypercapnia. Recently, a series of clinical conditions that assist in decision-making regarding orotracheal intubation have been suggested, and these include signs of unsustainable respiratory work and the development of respiratory acidosis [17]. In COVID-19, clinical deterioration seems different from that observed in ARDS secondary to bacterial pneumonia, where the evolution of radiological involvement and hypoxemia leads to the development of respiratory alkalosis (hypocapnia) [18], [19], [20]. Its clinical correspondence, represented by the increase in respiratory rates (superficial tachypnea), could be a turning point when evaluating the need for orotracheal intubation. Simply put, in the early stages, there is limited evidence of excess functional dead space, as observed in classical ARDS [21]. The possibility of embolic phenomena in the pulmonary circulation [22] or perfusion deficits [6] caused by microangiopathy in SARS-CoV-2 infection could be responsible for the increase in dead space at a microscopic level.

In many cases, absence of a significant impairment in pulmonary compliance and resistance, evidenced in ventilator mechanics and parameters, has been reported [23]. Moreover, the presence of severe refractory hypoxemia with the absence of peak and high plateau pressures support the notion that COVID-19 presents differently from typical ARDS [7]. Therefore, there is no evidence supporting the benefit of prone positioning after intubation in achieving significant improvement in oxygenation through it hemodynamic impact; further, prolonged periods of sedation at high doses and the use of muscle relaxants are required [10,24]. It may result in increased durations of mechanical ventilation and stay in the intensive care unit (and therefore increase the possibility of infectious complications), difficulty in achieving enteral nutrition, and induce the use of renal replacement techniques [25]. In refractory hypoxemia, in patients who undergo mechanical ventilation with markedly decreased pulmonary compliance and increased nonaerated lung regions, the effectiveness of the prone position could be debatable as nonaerated lung areas are not totally recruitable. However, the prone position could be beneficial in awake patients with spontaneous breathing as additional therapeutic effort is not required [26].

Finally, involvement of the microcirculation in the viral infection, resulting in compensatory pulmonary vasoconstriction, can lead to so-called hypoxic vasoconstriction [27,28], with intrapulmonary blood flow redistribution [29,30]. In this scenario of mismatch [31], a benefit can be obtained with inhaled nitric oxide at concentrations lower than 20 ppm (or isoproterenol) as early rescue therapy [32], [33], [34] in case of refractory hypoxemia. This is supported by the management of gas exchange in infants with acute viral bronchiolitis on mechanical ventilation [35].

## Nonhypoxic hypoxemia

Severe hypoxemia (partial pressure of oxygen in the arterial blood between 60 and 40 mmHg) and hypoxia (decreased oxygen diffusion in tissues and cells) are concepts that are usually correlated (hypoxic hypoxemia), although hypoxia may be caused by other processes.

It is common when assessing patients with COVID-19 to observe a dissociation between the absence of clinical manifestations and the detection of moderate/severe hypoxemia through arterial blood gas values. Together with the presence of diffuse opacities observed in radiological studies, these findings usually indicate the need for hospital admission. Hypoxemia (demonstrated by measuring the partial pressure of oxygen [PaO_2_] in arterial blood) that does not lead to significant hypoxia is difficult to explain based on usual pathophysiological mechanisms [36], but it may be observed if lung compliance is maintained.

The treatment sequence for COVID-19 starts with the administration of oxygen to patients with hypoxemic respiratory failure. Initially, supplemental oxygen is administered through a nasal cannula or Venturi mask, regardless of the absence of associated clinical symptoms. Subsequently, the concentration of inhaled oxygen is gradually increased until reservoir masks or HFNCs are required, and the progression of pulmonary involvement is verified by radiological controls. Despite the risks of bioaerosol transmission, several reports [37,38] have suggested that HFNCs do not confer a higher risk of bioaerosol dispersion than conventional oxygen masks. Finally, patients may present an increase in respiratory work, presenting as tachypnea and respiratory alkalosis. Dyspnea usually occurs when PaO_2_ is severely affected. In clinical practice, the appearance of a state of anxiety and agitation is always associated with the development of tachypnea. The worsening of the respiratory pattern associated with refractory hypoxemia despite the increase in the inspired fraction is usually the point at which evaluation is requested to decide whether one should proceed with rapid-sequence intubation.

In patients with low pulmonary compliance, inspiratory effort is associated with low tidal volume and increased respiratory rates [18,39,40]. However, patients with normal compliance do not feel the inspiratory effort and thus start with increased ventilation without an increase in inspiratory transpulmonary pressure. For a given pressure per breath (Per), the perception of effort is a function of maximal inspiratory pressure (MIP). Therefore, the greater is the Per/MIP ratio, the greater is the perception of respiratory effort, which explains the coexistence of hypercapnia and hypoxia. In these patients, changes in CO_2_ may induce dyspnea independently from inspiratory effort; indeed, hypoxemia is a very weak stimulus for dyspnea.

CO_2_ solubility is approximately 20 times that of O_2_. This is a major reason for why patients with pneumonia (as occurs with COVID-19) have difficulty diffusing oxygen into the blood but are still able to readily diffuse CO_2_ out of their blood. Silent hypoxemia as a physiologic phenomenon does not appear to be COVID-19-specific. Rather, it seems to follow established physiological principles.

A high oxygen concentration (and low nitrogen concentration) may induce toxicity, with individual susceptibility [41,42]. This lung toxicity is known to induce oxidative processes and cause diffuse alveolar damage, epithelial injury in distal airways, and finally, injury to the capillary endothelium [43]. There is some similarity between endothelial injury and the endotheliitis caused by SARS-CoV-2 infection [44]. In this scenario, what concentration of supplemental oxygen should be administered for silent or asymptomatic hypoxemia? If a risk–benefit balance is considered, this decision is difficult from a medical point of view, but avoiding overtreatment is the best course of action from a logical standpoint.

How can the phenomenon of nonhypoxic hypoxemia be explained? A question worth asking is whether the PaO_2_ values obtained from arterial samples can be influenced by some kind of physiological conditions. If we analyze the dissociation that also occurs between peripheral oxygen saturation and the degree of hypoxemia, oximetry-estimated saturation (SpO_2_) can differ from true arterial oxygen saturation, mainly at an SpO_2_ of 95% [45]. However, hypoxia occurs only with PaO_2_ values <40 mmHg (SpO_2_ of approximately 75%), and when oxygen extraction increases, normal oxygen consumption is maintained [45]. Therefore, it makes sense to provide high concentrations of supplemental oxygen in this context, despite the risk of worsening endothelial damage.

Areas of uncertainty exist. For example, does the SARS-CoV-2 infection trigger an alteration in the oxygen transport by hemoglobin? Could it be related to hypoxic vasoconstriction phenomena? Does noninvasive ventilation [20,46] induce ventilator-induced lung injury and contribute to a worse outcome? What is the effect of inhaled pulmonary vasodilators such as nitric oxide? When should intubation be performed?

## Phenotypes

Given the great variability of clinical presentation, different clinical phenotypes associated with COVID-19 have been defined [10] to drive the therapeutic strategies to consider, ranging from benign forms without lung (or hypoxemia) involvement that require only symptomatic treatment (phenotype 1) to evolutionary forms of radiological involvement and higher degrees of hypoxemia that usually require hospitalization (phenotypes 2 and 3), and to the most severe clinical forms characterized by the need for invasive mechanical ventilation (phenotypes 4 and 5) with differences in ventilatory parameters and rescue measures to consider [10]. Interestingly, silent hypoxemia was not reported in this reference.

## How to manage at the bedside?

The two most important strategies to consider when deciding whether endotracheal intubation is required are respiratory observation and continuous SpO_2_ monitoring. Difficulties in endotracheal intubation should be anticipated. A hallmark of COVID-19 is the rapid development of refractory hypoxemia with a poor response to oxygen supplementation, suggesting intrapulmonary shunting. This can overwhelm local resources, resulting in shortages of trained staff, oxygen supplies, and ventilators [47]. Local guidelines can help clinicians in making these difficult decisions with patients and their relatives. It is interesting to remember that oxygen needs to increase by a factor of 2.6 if the target SpO_2_ is 93% (instead of 90%) and by a factor of 8.5 if the target SpO_2_ is 98%. Thus, the titration of oxygen concentration for achieving an SpO_2_ of 90% has been implemented for COVID-19 management in regions where oxygen shortages were documented.

Lastly, we schematically outline the following temporal sequence of measures for respiratory management depending on the increase in clinical severity:

(1) In patients with clinically well-tolerated mild to moderate hypoxemia (silent hypoxemia), regardless of the extent of pulmonary opacities, the administration of supplemental oxygen therapy is required. Provided that SpO_2_ remains above 80%, no hypoxia manifestations should be expected. Thus, maintaining SpO_2_ values lower than those usually indicated to start supplemental oxygen administration (93%) should be considered if they are clinically well tolerated.
(2) Patients with symptomatic moderate to severe hypoxemia could benefit from the administration of supplemental oxygen through reservoir masks or HFNCs, although no evidence of outcome improvement with CPAP systems has been reported [48]. Awake prone positioning may be tried [49]. Patients with SARS-CoV-2 infection often present with volume depletion due to fever and tachypnea; the initial goal should be to correct hypovolemia, with careful attention to water balance. In addition, mucolytics may impede airway obstruction [50].
(3) The decision of intubation and mechanical ventilation should not be made based on a single isolated parameter, such as hypoxemia severity or the extent of pulmonary opacities observed in imaging tests. If required, it should be performed by the most skilled available operator after preoxygenation and rapid-sequence induction of sedation using neuromuscular blockade with succinylcholine or rocuronium [51,52]. Late HFNC or CPAP failure may be harmful, with late intubation being associated with life-threatening complications during the procedure.

Thus, intubation should be indicated in conjunction with the degree of clinical involvement documented by the development of any of the following conditions:

(1) Subjective patient sensation of physical exhaustion and sensation of nervousness or anxiety associated with difficulty to clear secretions and refractory hypoxemia.
(2) Appearance of severe tachypnea and its analytical translation represented by increasing respiratory alkalosis and the patient being unable to maintain SpO_2_ above 85%.
(3) Deterioration of clinical status (represented by progression of tachypnea with increase in the work of breathing, presenting with the use of accessory respiratory muscles and persistence of low SpO_2_) despite supplemental oxygen therapy with reservoir mask, a HFNC, or CPAP systems.
(4) Encephalopathy with significant alteration of the level of consciousness, with development of hypercapnia or acidemia.
(5) Progression to multiple organ failure/dysfunction.
(6) Severe myocardial dysfunction.

## Conclusions

In summary, silent hypoxemia is common in patients with SARS-CoV-2 infection. The risk of sudden respiratory arrest during an emergency intubation, which could expose healthcare workers to infection, should be considered along with the risks of premature intubation. Careful monitoring of respiratory status to identify whether intubation is required is the main goal. Criteria for intubation need to be revisited based on updated evidence showing that many patients with severe hypoxemia do not experience increased work of breathing. This has some implications in patient management and may explain in part the broad differences in outcomes reported among intubated patients.

## Conflicts of Interest

The authors declare that they have no known competing financial interests or personal relationships that could have appeared to influence the work reported in this paper.
